# Perceptions of dental students on the integration of artificial intelligence in radiology clinical education

**DOI:** 10.3389/fdmed.2025.1735299

**Published:** 2026-01-02

**Authors:** Ricky Amreek Suri, Chiraag Gohel, Wazeer Alghamdi, Brandon Crowther, A. Isabel Garcia, Anita Gohel

**Affiliations:** 1 Oral & Maxillofacial Radiology Resident, University of Florida College of Dentistry, Gainesville, FL, United States; 2 Department of Biostatistics and Bioinformatics, Computational Biology Institute, The George Washington University, Washington, DC, United States; 3 University of Florida College of Dentistry, Gainesville, FL, United States; 4 Oral and Maxillofacial Diagnostic Sciences, Oral and Maxillofacial Radiology, University of Florida College of Dentistry, Gainesville, FL, United States; 5 The School of Dentistry, The University of Jordan, Amman, Jordan

**Keywords:** artificial intelligence, automation bias, curriculum development, dental education, radiology

## Abstract

**Objective:**

To assess dental students' perceptions of artificial intelligence (AI) in radiology education, focusing on diagnostic value, curriculum preparedness, and faculty support.

**Methods:**

An anonymous survey was administered to third-year dental students (*n* = 66, response rate 71.7%) at the University of Florida College of Dentistry after exposure to the Overjet Caries Assist (OCA) platform (Overjet Inc. Claymont, DE, USA). Likert-scale, multiple-choice, and open-ended items captured attitudes toward diagnostic accuracy, skill development, curriculum integration, and patient communication. Descriptive statistics, polychoric correlations with bootstrap resampling, and thematic analysis of qualitative responses were conducted.

**Results:**

Most students reported that AI improved their ability to detect caries (89.4%) and enhanced radiographic interpretation (92.4%). However, only 16.7% agreed the curriculum adequately prepared them to use AI clinically, and just 45.5% felt confident about integrating AI into future practice. Open-ended feedback highlighted three themes: 1) need for structured faculty training, 2) earlier and more frequent AI exposure, and 3) emphasis on mitigating automation bias, or the over reliance on technology and automated systems in clinical judgement. Correlation analysis revealed strong associations between improved interpretation, skill development, and patient communication (r > 0.80), however, significant negative correlations emerged between student outcomes and perceptions of faculty preparedness.

**Conclusions:**

Students value AI as a diagnostic learning aid but identify gaps in curricular structure and faculty calibration. A structured, faculty-led AI curriculum introduced early in training and paired with patient communication strategies may optimize preparedness while safeguarding critical thinking.

## Introduction

Artificial intelligence (AI) is rapidly becoming a cornerstone of modern healthcare, particularly in diagnostic imaging (1). In dentistry, AI can assist in detecting caries, identifying common clinical findings as in support of a radiographic interpretation, and improving diagnostic consistency (1–3). As the dental field progresses, educators must assess whether students are adequately prepared to integrate these tools into clinical practice.

The integration of artificial intelligence (AI) into dentistry is transforming both clinical practice and dental education. The recent developments in AI within the healthcare system has made it increasingly evident that dentists and dental students need a comprehensive and analytic education in AI in order to critically assess new technologies and their applications in clinical practice. There is a growing consensus that dental and health professions curricula should incorporate foundational knowledge of artificial intelligence to prepare future clinicians for its expanding role in clinical decision-making (4, 5). It is essential for dentists to understand and appreciate how AI can improve patient care and communication. As AI continues to evolve, tools such as AI-driven caries detection systems are being introduced in both clinical and academic settings. Although studies in medical education report positive student attitudes toward AI modules (6–9), data remains limited on how dental students perceive and integrate AI-assisted tools within clinical curricula (4, 10, 11).

The University of Florida College of Dentistry recently integrated the Overjet software into the dental curriculum. Overjet is a computer vision artificial intelligence platform, with deep-learning modules that segment bitewing and periapical radiographs, highlighting potential carious lesions, classifying them as preventive or restorative, measures alveolar bone levels, and identifies periapical radiolucencies. Overjet also offers an AI-powered training module specifically designed to help dental students develop diagnostic skills in caries detection. The Overjet Caries Assist (OCA) device and the Overjet training module have been used to train third-year dental students to enhance their radiographic diagnostic skills. This study investigates dental students' perceptions of AI's role in their education, the perceived learning curve, and the level of support provided by the curriculum and faculty.

## Materials and methods

### Study design and participants

This study was reviewed by the Institutional Review Board (IRB) at University of Florida (Protocol # ET00047431) and was determined to be exempt. An anonymous, cross-sectional survey was distributed to all 92 third-year dental students enrolled in the Spring 2025 radiology course. Sixty-six students completed the survey (response rate 71.7%). Participation was voluntary and uncompensated. All students were provided with the same structured exposure through a required radiology laboratory session and received an identical standardized exercise with the Overjet Caries Assist training module. The radiology faculty and residents were calibrated prior to the learning activity to ensure consistency in instructional guidance.

### Survey instrument

The survey items were adapted from prior studies evaluating student attitudes toward AI in medical education and were reviewed by two experts in oral and maxillofacial radiology and one expert in dental education to ensure content validity (12). The survey (Appendix 1) included:

1. Eight Likert-scale items (1 = Strongly Disagree to 5 = Strongly Agree) on diagnostic impact, skill development, curriculum preparedness, faculty support, and future confidence.
2. One multiple-choice item on the perceived AI learning curve.
3. Open-ended prompts for reflections and recommendations.

The results were downloaded as a comma-separated value (CSV) file. The mean, standard deviation, median, minimum, and maximum score was analyzed. Quantitative data were analyzed descriptively. Agreement was defined as ratings of 4–5, disagreement as 1–2, and neutrality as 3. Reliability was assessed via Cronbach's alpha. Polychoric correlations with bootstrap resampling (100 iterations) were computed to examine associations among ordinal variables. Two-tailed *p*-values were computed to test whether correlations significantly differed from zero. For each correlation, the *p*-value was calculated as twice the proportion of bootstrap samples where the correlation coefficient had the opposite sign from the observed correlation. 95% confidence intervals were calculated using the 2.5th and 97.5th percentiles of the bootstrap distribution for each correlation coefficient. Standard errors for each correlation coefficient were calculated as the standard deviation of the bootstrap distribution. Given the multiple correlations tested (28 unique pairs from the 8 variables), the Benjamini-Hochberg false discovery rate (FDR) correction was applied to control for multiple comparisons. Adjusted *p*-values (q-values) were calculated, with statistical significance evaluated at *q* < 0.05.

Qualitative responses (*n* = 95) underwent thematic content analysis to identify recurring concerns and recommendations. Thematic analysis is the process of analyzing qualitative data to identify and interpret recurring patterns.

## Results

### Quantitative findings

The internal consistency of the eight-item Likert scale was assessed using Cronbach's alpha, resulting in a coefficient of *α* = 0.835. This value exceeds the commonly accepted threshold of 0.70, indicating good reliability of the survey instrument. Descriptive statistics, including the mean, standard deviation, median, minimum, and maximum scores, are presented in Table 1.

**Table 1:** Summary metrics for survey item responses.

Question	Mean	Std Dev	Median	Min	Max
1. The integration of dental AI tools has enhanced my ability to detect and diagnose dental caries accurately.	4.38	0.82	5	1	5
2. Using AI-assisted learning has improved my understanding of radiographic interpretation of common dental findings.	4.38	0.82	5	1	5
3. The presence of AI tools has affected my development of traditional clinical skills and critical thinking.	4.12	0.97	4	1	5
4. AI tools have influenced how I communicate clinical findings such as dental caries to patients.	4.32	1.10	5	1	5
5. The integration of AI will affect patient trust and acceptance of my diagnostic recommendations.	4.48	0.81	5	1	5
6. The dental school curriculum effectively prepared me to use AI tools in clinical practice.	3.05	0.89	3	2	5
7. Faculty members were knowledgeable and supportive in guiding AI tool usage.	3.08	1.00	3	1	5
8. I feel confident in my ability to integrate AI tools into my future dental practice.	3.59	0.98	3	2	5

**Figure 1:**
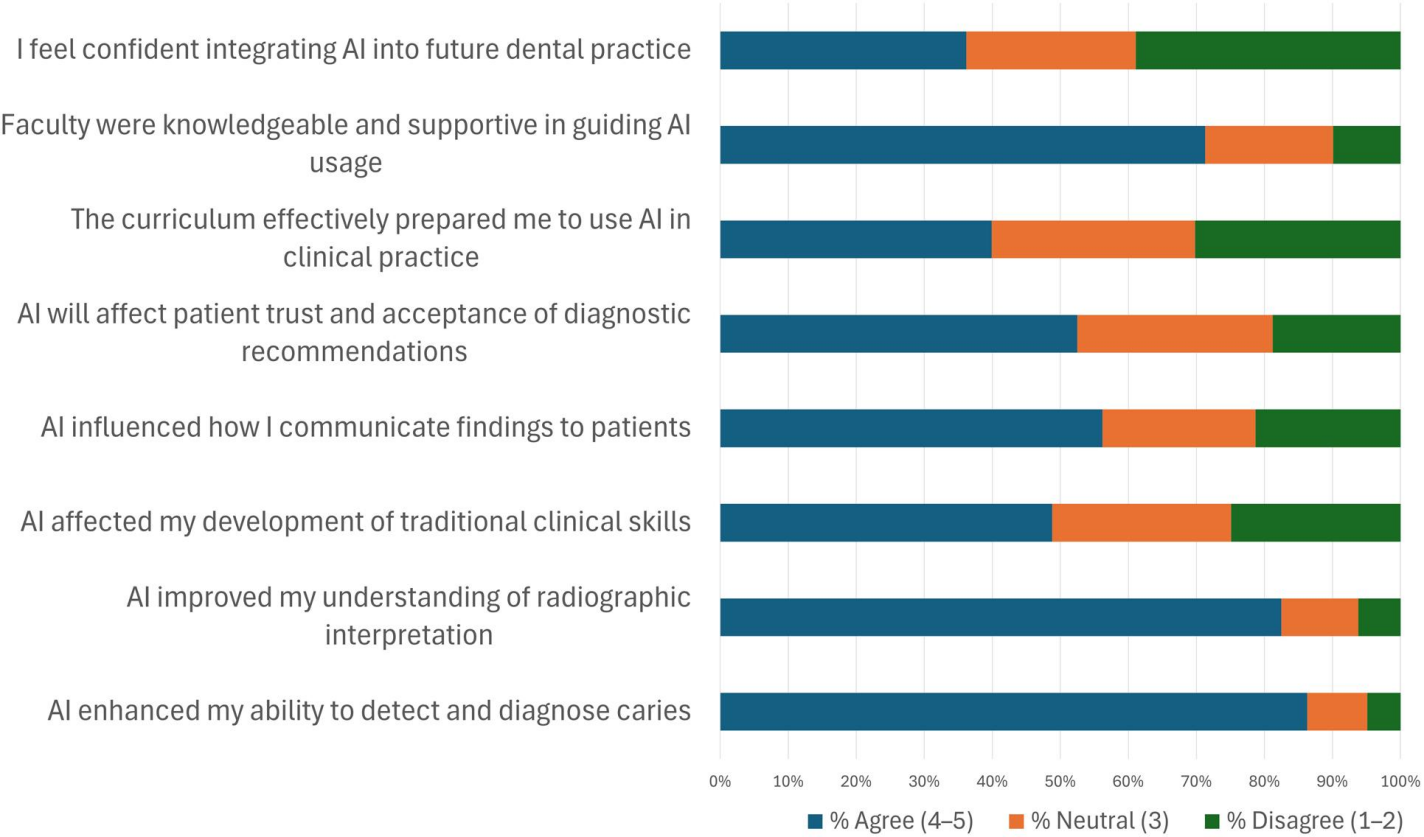
Distribution of agreement levels (%) across AI-related survey domains. Each color corresponds to student responses to statements about AI in dental radiology education. (Blue-Agree; Orange-Neutral; Grey-Disagree).

A substantial majority of students (89.4%) agreed or strongly agreed that AI tools enhanced their ability to detect and diagnose dental caries. Similarly, 92.4% reported that AI-assisted learning improved their understanding of radiographic interpretation of common findings. However, concerns regarding the impact of AI on traditional clinical skill development were evident: 81.8% expressed awareness that AI might hinder their skill acquisition, while 4.6% disagreed, and 13.6% remained neutral. Over three-quarters of the respondents (77.3%) felt that AI tools improved clarity in patient interactions, whereas 16.7% were neutral, and 6.0% disagreed (Figure 1).

Only 16.7% of students believed that the current curriculum effectively prepared them to utilize AI tools in clinical practice. While students tended to view faculty support for AI integration unfavorably, survey results showed that most held neutral positions on whether the faculty were knowledgeable and supportive. A total of 24.2% of respondents rated faculty as knowledgeable and supportive in guiding AI usage, with the majority (47.0%) being neutral, and 28.8% disagreeing. Accordingly, confidence in integrating AI into future clinical practice remained divided, with only 45.5% expressing high confidence, 43.9% being neutral, and 10.6% reporting a lack of confidence (Figure 1).

**Figure 2:**
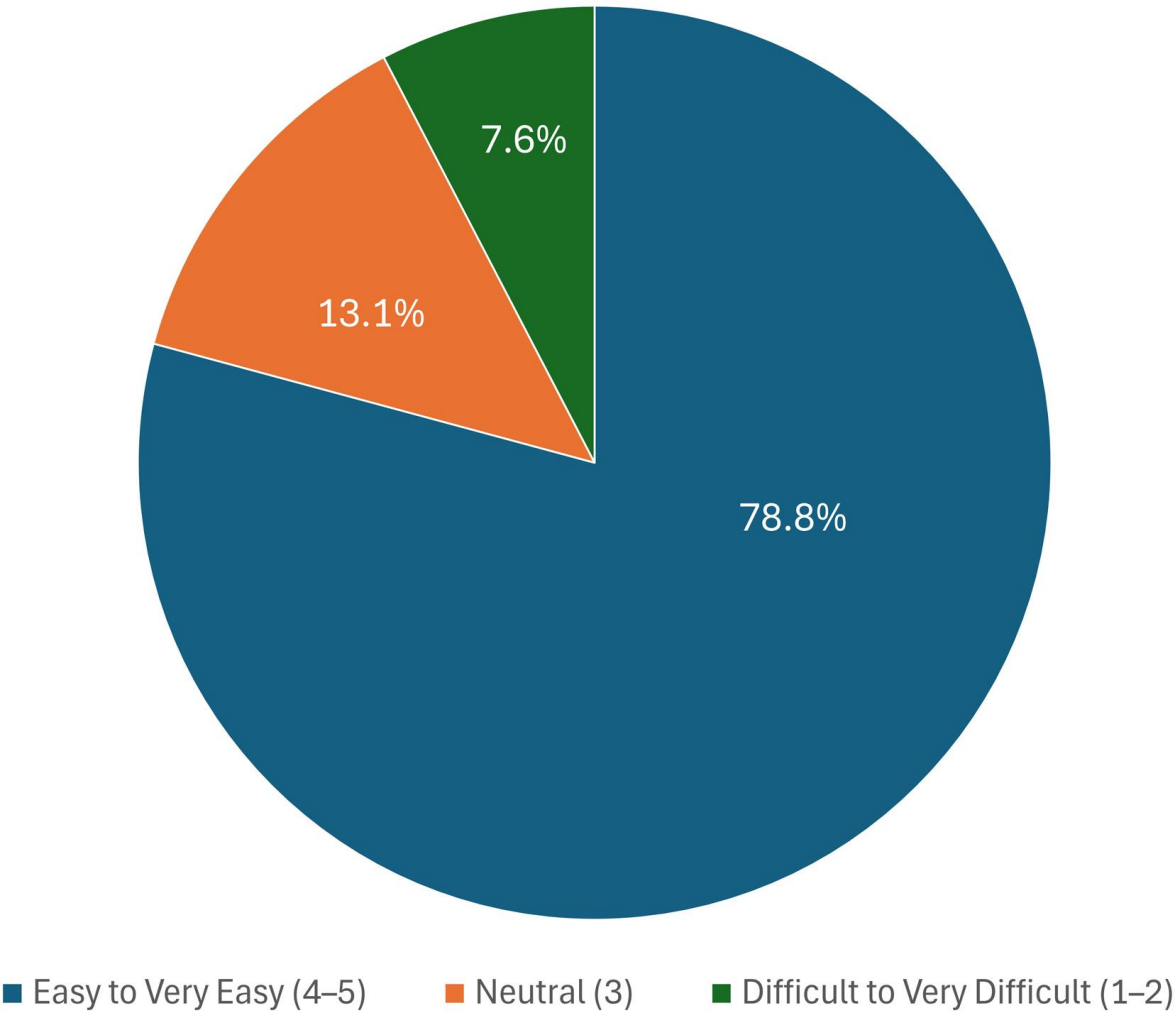
Survey breakdown of AI learning curve perceptions (blue-easy; orange-neutral; grey-difficult).

Regarding the learning curve, 78.8% of students described AI tools as “Easy” or “Very Easy” to use, particularly when supported by residents or faculty. Meanwhile, 13.6% were unsure, and 7.6% found the tools “Difficult” or “Very Difficult” to navigate (Figure 2).

### Correlation analysis results

**Figure 3:**
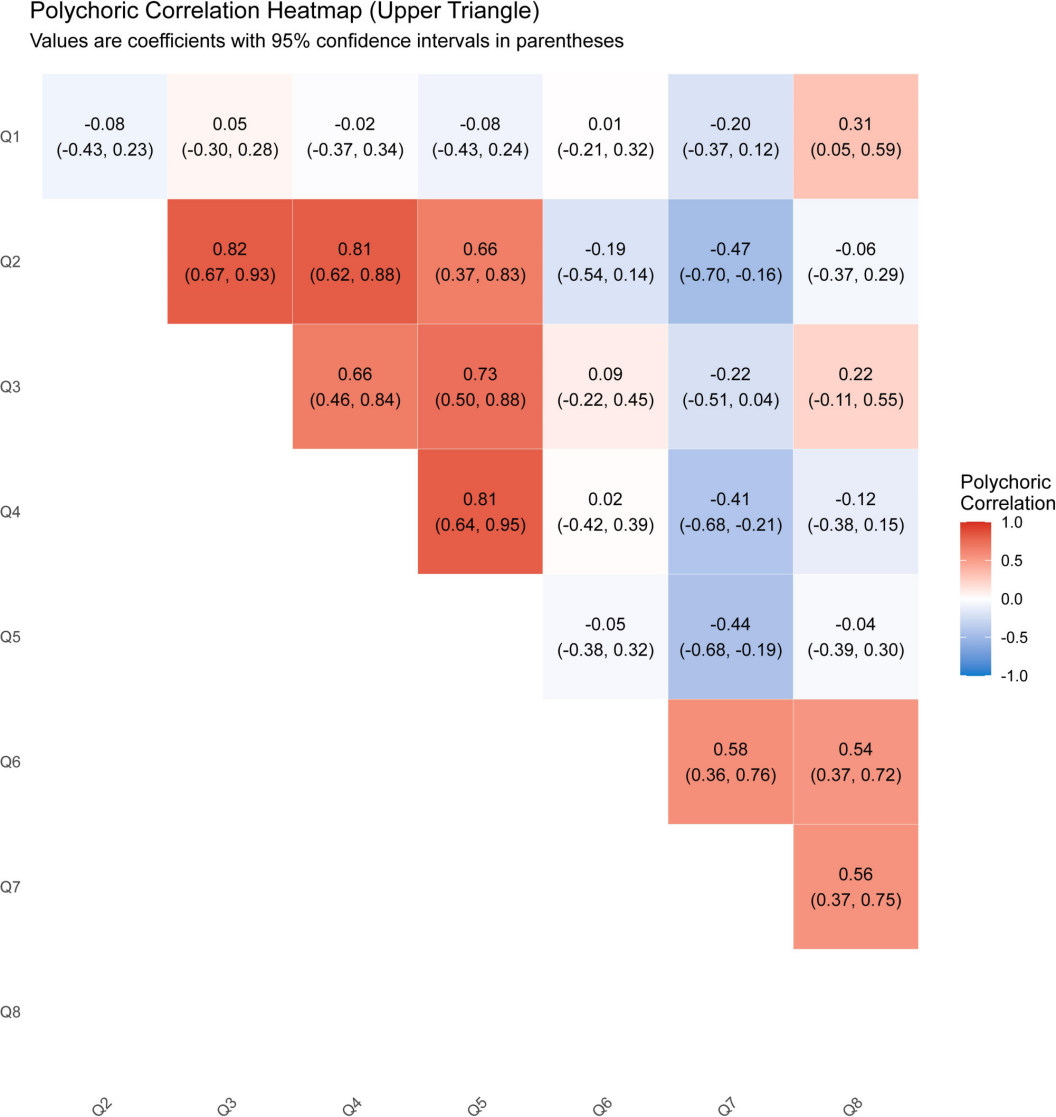
Polychoric correlation heatmap (upper triangle). Values are coefficients with 95% confidence intervals for survey questions 1-8 (Q1-Q8). (Colors: Red = positive correlation; Blue = negative correlation; White = near zero correlation).

Polychoric correlation analysis with bootstrap confidence intervals was conducted to examine relationships among survey items. After applying the Benjamini-Hochberg false discovery rate (FDR) correction for multiple comparisons, 13 out of 28 possible item pairs remained statistically significant at a threshold of q < 0.05 (Figure 3).

### Strong positive correlations

Several strong and statistically significant correlations were observed:

- Radiographic interpretation and clinical skill development: AI-assisted learning that improved radiographic interpretation (Q2) was strongly correlated with perceptions that AI influenced clinical skill development (Q3), r = 0.82, 95% CI [0.67, 0.93], q < 0.001.
- Radiographic interpretation and patient communication: AI-assisted learning (Q2) also showed a strong correlation with the influence of AI on patient communication (Q4), r = 0.81, 95% CI [0.62, 0.88], q < 0.001.
- Patient communication and trust: The perceived impact of AI on patient communication (Q4) was strongly associated with expectations of AI’s effect on patient trust and acceptance (Q5), r = 0.81, 95% CI [0.64, 0.95], q < 0.001.

### Moderate positive correlations

Moderate but significant correlations included:

- Clinical skill development and patient trust: r = 0.73, 95% CI [0.50, 0.88], q < 0.001.
- Clinical skill development and patient communication: r = 0.66, 95% CI [0.46, 0.84], q < 0.001.
- Radiographic interpretation and patient trust: r = 0.66, 95% CI [0.37, 0.83], q < 0.001.

### Institutional support correlations

Correlations related to institutional preparedness and faculty support were also significant:

- Curriculum preparedness and faculty support: r = 0.58, 95% CI [0.36, 0.76], q < 0.001.
- Faculty support and student confidence: r = 0.56, 95% CI [0.37, 0.75], q < 0.001.
- Curriculum preparedness and student confidence: r = 0.54, 95% CI [0.37, 0.72], q < 0.001.

### Significant negative correlations

Interestingly, several negative correlations were identified, suggesting potential disconnects between perceived faculty support and other AI-related benefits:

- Radiographic interpretation and faculty support: r = −0.47, 95% CI [−0.70, −0.16], q < 0.001.
- Patient trust and faculty support: r = −0.44, 95% CI [−0.68, −0.19], q < 0.001.
- Patient communication and faculty support: r = −0.41, 95% CI [−0.68, −0.21], q < 0.001.

### Weak but significant correlation

A weak yet statistically significant correlation was found between diagnostic enhancement and confidence in AI integration:

- Caries detection and confidence in AI integration: r = 0.31, 95% CI [0.05, 0.59], q < 0.001.

### Qualitative themes

The analysis of 95 open-ended qualitative responses revealed that students are generally receptive to the integration of AI in dental education, while also identifying several areas in need of improvement (Table 2). Three principal themes emerged from the data:

1. Faculty training and calibration: Students noted inconsistent faculty guidance and recommended workshops for faculty training and calibrations.
2. Early and frequent exposure: Calls for AI integration in preclinical labs to normalize use.
3. Mitigating automation bias: Emphasis on critical thinking and avoiding over-reliance on AI.

**Table 2:** Thematic summary of student feedback on AI in dental education.

Theme	Sentiment	Frequency
More integration/training needed	Suggestive	High
Faculty preparedness inconsistent	Concern	Medium
Positive engagement with AI	Positive	Medium
Overreliance/critical thinking	Concern	Low
Learning curve experience varied	Neutral	Medium
Specific suggestions for curriculum	Suggestive	Medium

Many students viewed AI positively, describing their experiences as exciting and valuable, particularly in enhancing diagnostic thinking. However, there were concerns about overreliance on AI and its potential impact on the development of traditional clinical reasoning skills.

## Discussion

The findings of this study highlight a generally positive perception among dental students regarding the integration of AI tools into their diagnostic training, particularly in the context of caries detection and radiographic interpretation. A substantial majority (89.4%) reported that AI enhanced their ability to detect and diagnose dental caries, and 92.4% indicated improved understanding of radiographic findings. These results align with existing literature that suggest that AI can serve as a valuable adjunct in dental diagnostics by increasing accuracy and reinforcing visual pattern recognition skills (3, 13, 14).

Despite these benefits, the majority of the respondents (81.8%) expressed concern that reliance on AI might hinder the development of foundational clinical skills. This awareness reflects a broader tension in health professions education—while AI can augment learning, there is a risk that over-reliance may compromise the cultivation of independent diagnostic reasoning (15, 16).

The diagnostic advantages perceived by the students in our study reflect the known capabilities of AI in oral and maxillofacial radiology. Heo et al. (2021) demonstrated that AI tools are already capable of assisting with caries detection, lesion classification, and periodontal bone level measurement, emphasizing the technical feasibility of using AI tools. However, our findings reveal that technical potential does not necessarily translate into educational preparedness, as students reported gaps in learning and curricular integration (17).

A strong majority of respondents (77.3%) reported that AI tools enhanced clarity in patient communication, indicating broad support for their use in clinical interactions. However, a smaller subset remained neutral (16.7%) or disagreed (6.0%), suggesting that some variability persists. This is potentially due to inconsistent implementation or limited training on how to integrate AI outputs into patient-provider dialogue. These findings support the need for structured guidance on using AI as a communication aid, complementing its diagnostic utility. Only 16.7% of students reported feeling that the curriculum adequately prepared them to use AI in clinical practice. This gap in perceived preparedness suggests that although students are exposed to AI tools, they may lack the formal instruction and contextual understanding needed to apply them confidently and effectively. Confidence in integrating AI into future practice was modest (45.5%), and an even smaller proportion of students (24.2%) viewed faculty as knowledgeable and supportive. While some faculty engagement may exist, these figures indicate that it has not yet translated into structured, competency-based learning experiences. The University of Florida College of Dentistry is among the first dental institutions to introduce AI-assisted radiographic diagnosis. However, the initial implementation was not integrated into the electronic dental record (EDR) and was not accessible to all faculty—factors that likely influenced students' perceptions. These findings are consistent with prior research highlighting gaps in formal AI education and training, which may hinder the full realization of its potential in medical radiology (18). Since the time of the survey, a more structured process for faculty training and engagement has been established, and Overjet software has now been fully integrated with the EDR.

Interestingly, several significant negative correlations emerged between faculty support and students' perceived benefits of AI in radiographic interpretation, patient communication, and trust. These patterns may indicate a disconnect between faculty guidance and students' expectations or experiences with AI tools. A likely explanation is that limited and inconsistent faculty engagement with AI technology reduced students' confidence in its effectiveness. At the time of the survey, the image-uploading process for the AI-assisted program was cumbersome and not well streamlined, and access to the software was restricted to only a few faculty members. Together, these factors may have reinforced students' perceptions that the system was difficult to use, contributing to the negative associations observed. Since the survey, all faculty members have been granted access to the AI software, which is expected to improve both engagement and student perceptions.

Optimistically, a substantial majority of students (78.8%) reported that the AI learning curve was “Easy” or “Very Easy,” particularly when supported by residents or faculty. This indicates that with appropriate guidance, students can adopt AI tools with minimal technical difficulty. These findings reflect a growing acceptance of AI in dental education. However, this optimism is tempered by concerns about skill development, communication, and curricular preparedness. To fully realize the potential of AI in clinical training, dental schools must address these concerns through targeted curriculum enhancements, enhanced faculty development, and structured student support mechanisms.

Given the ordinal structure of the Likert scale data, polychoric correlations were computed to examine relationships between survey questions. This method is well-suited for analyzing associations between ordinal variables, as it estimates the correlation between underlying continuous variables that have been categorized into ordinal scales (19, 20). The correlation analysis revealed several statistically significant associations that yield important insights into students' perceptions of AI integration within dental education.

The strongest positive correlations emerged between AI-assisted radiographic interpretation and its perceived impact on both clinical skill development and patient communication. These findings suggest that students who recognize the diagnostic utility of AI also tend to recognize its broader educational and communicative benefits, indicating a holistic appreciation of AI's multifaceted role in clinical training. The robust association between AI's influence on patient communication and its anticipated effects on patient trust and acceptance emphasizes the importance of communication-centered AI applications in dental practice. As dental professionals increasingly integrate AI tools into diagnostic and treatment workflows, the capacity to articulate AI-informed insights to patients may become an integral component of patient-centered care (21).

Moderate correlations between clinical skill development and patient trust further emphasize the interconnectedness of technical proficiency and interpersonal dynamics. Students who view AI as enhancing their clinical capabilities are more likely to anticipate positive patient responses, suggesting that confidence in AI may translate into greater trust in its clinical application. Institutional support variables also reveal meaningful correlations. Perceptions of curriculum preparedness were positively associated with both faculty support and student confidence in integrating AI into practice. These findings highlight the pivotal role of structured educational frameworks and informed faculty engagement in fostering student readiness for AI adoption. Nevertheless, the relatively low overall confidence levels reported by students suggest that current curricular efforts may be insufficient, and further investment in AI-focused training is warranted (1, 11).

Several significant negative correlations emerged between faculty support and students' perceived benefits of AI in radiographic interpretation, patient communication, and trust. These patterns may indicate a disconnect between faculty guidance and students' expectations or experiences with AI tools. A likely explanation is that limited and inconsistent faculty engagement with AI technology reduced students' confidence in its effectiveness. Earlier in the Discussion, we noted that limited and inconsistent faculty access to the AI platform, as well as a cumbersome image-uploading process likely contributed to students’ negative perceptions of faculty support. These same logistical barriers may also explain the significant negative correlations observed, underscoring the importance of improving system accessibility and workflow efficiency. Since the survey, all faculty members have been granted access to the AI software, which is expected to improve both engagement and student perceptions.

Negrete et al. (2025) further highlight the need for educational frameworks to embed AI into radiology training. The topics identified in our qualitative analysis including faculty calibration, early exposure, and mitigation of automation bias, echo these recommendations suggesting that dental education must adapt to ensure students are not only exposed to AI but also trained to critically and ethically integrate it into routine clinical practice (22).

Finally, a weak but significant correlation between enhanced caries detection and confidence in AI integration suggests that while students recognize specific diagnostic advantages, this does not necessarily translate into broader confidence in clinical implementation. This gap may reflect uncertainty about the practical, ethical, or procedural aspects of AI use in real-world settings.

The analysis of qualitative themes revealed that students are generally receptive to the integration of AI in dental education but emphasized several areas for improvement. A prominent theme was the need for more comprehensive integration and training, with students expressing a desire for earlier and more consistent exposure to AI tools across the curriculum with suggestions for improvement included clearer tutorials, faculty standardization, and collaborative learning opportunities. This points to a future research opportunity that can be directed towards a multidisciplinary approach to curriculum redesign to support optimal AI adoption in dental education (5). A majority of students viewed AI positively, and described their experiences as exciting and valuable, particularly with respect to improving diagnostic thinking. However, there were concerns about overreliance on AI and its potential impact on the development of traditional clinical reasoning skills. Collectively, these themes underscore the importance of structured, well-supported AI integration to maximize its educational benefit. The students emphasized the importance of mitigating automation bias by fostering critical thinking and discouraging over-reliance on AI systems in clinical decision-making. Rather than replacing clinical judgment, AI is expected to serve an assistive role in supporting diagnosis and patient care, an approach best described as Augmentative Intelligence (AuI) (23).

In the context of dental radiology, the integration of AI technologies is anticipated to enhance and augment dentists' diagnostic accuracy by combining technology with human intelligence, ultimately leading to better patient outcomes (24, 25).

Overall, these findings draw attention to the multifaceted nature of AI integration within dental education. Currently, there is a lack of accreditation standards regarding incorporation of AI and AuI into the dental curriculum. While students generally perceive AI as beneficial, particularly in the diagnostic and communicative domains, concerns remain about skill development, curriculum adequacy, and faculty alignment. Addressing these challenges through targeted curriculum enhancements, faculty development programs, and early exposure to AI tools may help bridge the gap between perceived benefits and practical confidence in AI adoption for diagnostic dentistry. Such efforts can equip future dentists with the requisite knowledge and competencies to deliver more optimal patient care (26).

## Limitations

This study is limited by its single-site design and a 71.7% response rate that may not generalize across other dental programs. At the time of the survey, we were unable to identify any other dental school that had incorporated Overjet AI into its curriculum; therefore, the study was necessarily conducted at a single site. Self-selection bias may have skewed the results toward students with a preexisting interest in AI. Future research should track objective skill acquisition longitudinally and examine patient-care outcomes. The term 'supportive' in the faculty-related survey item was not explicitly defined, and students may have had differing interpretations (e.g., encouragement vs. instructional assistance), which may introduce response variability.

## Conclusion

This study highlights the evolving role of artificial intelligence in dental education, particularly in diagnostic training and clinical preparedness. AI is poised to play a transformative role in dental diagnostics and dental education. As AI continues to reshape healthcare delivery, dental curricula must adapt to prepare future practitioners for ethical, effective, and confident use of these technologies. By addressing current limitations and leveraging student insights, institutions can cultivate a generation of clinicians who are both technologically proficient and clinically grounded. Despite generally positive perceptions of faculty support, students expressed low confidence in their ability to integrate AI into future practice, revealing gaps in curricular readiness. A structured, faculty-led integration, with emphasis on ethical use, critical thinking, and hands-on calibration, will enable students to utilize AI's potential safely and effectively. As AI becomes ubiquitous in dentistry, dental schools should embed AI into both preclinical and clinical instruction with structured modules, faculty engagement, and real-world scenarios to preserve competencies, optimize patient care, and prepare students for the transforming digital landscape of dental care in the future.

## Data Availability

The raw data supporting the conclusions of this article will be made available by the authors, without undue reservation.

## Appendix 1: Survey Form

Integration of Artificial Intelligence in Dental Education: Student Feedback Survey.

Please rate each statement on a scale of 1–5 (1 = Strongly Disagree, 5 = Strongly Agree).

Section 1: Educational Experience.

1. The integration of dental AI tools has enhanced my ability to detect and diagnose dental caries accurately.

□ 1 □ 2 □ 3 □ 4 □ 5

2. Using AI-assisted learning has improved my understanding of radiographic interpretation of common dental findings.

□ 1 □ 2 □ 3 □ 4 □ 5

3. The presence of AI tools has influenced the development of my traditional clinical skills and critical thinking.

□ 1 □ 2 □ 3 □ 4 □ 5

Section 2: Patient Care & Communication

4. AI tools have affected how I communicate clinical findings (e.g., dental caries) to patients.

□ 1 □ 2 □ 3 □ 4 □ 5

5. The integration of AI will influence patient trust and acceptance of my diagnostic recommendations.

□ 1 □ 2 □ 3 □ 4 □ 5

Section 3: Curriculum & Faculty Support

6. The dental school curriculum effectively prepared me to use AI tools in clinical practice.

□ 1 □ 2 □ 3 □ 4 □ 5

7. Faculty members were knowledgeable and supportive in guiding AI tool usage.

□ 1 □ 2 □ 3 □ 4 □ 5

8. I feel confident in my ability to integrate AI tools into my future dental practice.

□ 1 □ 2 □ 3 □ 4 □ 5

Section 4: Learning Curve

9. How would you rate the learning curve for using AI tools?

10. □ Very Easy □ Easy □ Not Sure □ Difficult □ Very Difficult

Section 5: Open Feedback

11. Approximately how long did it take you to feel comfortable using AI tools?

12. Please share any other thoughts about how AI has impacted your dental education:

13. Do you have any suggestions for improving AI integration in dental education?
